# Seroprevalence and risk factors of *Toxoplasma gondii* in Tibetan Sheep in Gansu province, Northwestern China

**DOI:** 10.1186/s12917-015-0358-0

**Published:** 2015-02-21

**Authors:** Ming-Yang Yin, Jin-Lei Wang, Si-Yang Huang, Si-Yuan Qin, Dong-Hui Zhou, Guang-Xue Liu, Qi-Dong Tan, Xing-Quan Zhu

**Affiliations:** State Key Laboratory of Veterinary Etiological Biology, Lanzhou Veterinary Research Institute, Chinese Academy of Agricultural Sciences, Lanzhou, Gansu province 730046 PR China; College of Veterinary Medicine, Hunan Agricultural University, Changsha, Hunan province 410128 RR China; College of Animal Science and Technology, Jilin Agricultural University, Changchun, Jilin province 130118 PR China; College of Animal Science and Technology, Anhui Agricultural University, Hefei, Anhui province 230036 PR China

## Abstract

**Background:**

*Toxoplasma gondii*, a protozoan parasite, infects almost all warm-blooded animals and humans. Limited information is available about *T. gondii* infection in Tibetan Sheep in Gansu province, northwestern China. In the present study, we estimated the seroprevalence and risk factors of *T. gondii* infection in this region of China.

**Results:**

A total of 1732 Tibetan Sheep were included from Tianzhu and Maqu in Gansu province. Antibodies to *T. gondii* were examined by modified agglutination test (MAT), and 352 (20.3%) out of 1732 Tibetan sheep were found positive. Multivariate logistic regression analysis was used to analyze the risk factors associated with seroprevalence, the results showed that age, gender, and numbers of past pregnancies were not the significant risk factors. However, Tibetan sheep in Maqu had a 1.64 times (odds ratio [OR] =1.637, 95% CI =1.291-2.075, *P* < 0.001) higher seroprevalence compared to Tianzhu, and the seropositivity in summer were 1.61 times (OR =1.608, 95% CI =1.122-2.303, *P* = 0.010) higher compared to Tibetan sheep in winter, followed by 1.42 times (OR =1.419, 95% CI =1.002-2.011, *P* = 0.049) in spring. Thus, season and location were considered as risk factors associated with *T. gondii* infection in this study.

**Conclusions:**

This is the first report of *T. gondii* seroprevalence in Tibetan sheep in Gansu province, which enriches the epidemiological data of *T. gondii* infection in Tibetan sheep in China. The results of this study indicate that Tibetan sheep in Gansu province are frequently exposed to *T. gondii*, posing a direct threat to the public health as well as to local sheep industry. These data is useful to strengthen future prevention and control of *T. gondii* infection in Tibetan sheep in this region.

## Background

*Toxoplasma gondii* is the most successful parasitic pathogen world-wide, infecting all warm blooded animals including humans [1,2]. Toxoplasmosis caused by *T. gondii* has been considered to be one of the major causes of abortion and neonatal mortality in sheep, thus, the infection is of great economic importance to the sheep industry [3]. Furthermore, humans can be infected by consuming undercooked meat, which is a risk factor for human health [4].

Seroprevalence of *T. gondii* in sheep have been reported extensively in different countries and the positive rates ranged from 3% to 95% [3]. In China, several studies indicated that the seroprevalence ranged from 4.4% to 29.8% in sheep [5-7]. Tibetan sheep, a specific species being cold and hypoxia resistant, mainly live in the alpine and pastoral areas, and they are an important economic resource for local people. Although two studies about the seroprevalence of *T. gondii* infection in Tibetan sheep were carried out in Tibet and Qinghai province [6,7], little information is available on the seroprevalence and risk factors for *T. gondii* in Tibetan sheep in Gansu province. Since Gansu province is one of the largest industrial regions for Tibetan sheep, it is important to quantify the prevalence of *T. gondii* infection in Tibetan sheep and further understand the potential risk posed to humans from this source of meat. This study was performed to determine the seroprevalence and risk factors for *T. gondii* infection in Tibetan sheep in Gansu province, northwestern China.

## Methods

### Ethics statement

The Tibetan sheep examined in the present study were handled in accordance with the Good Animal Practice requirements of the Animal Ethics Procedures and Guidelines of the People’s Republic of China. This study was approved by the Animal Ethics Committee of Lanzhou Veterinary Research Institute, Chinese Academy of Agricultural Sciences (Approval No. LVRIAEC2013-013).

### Animals and samples

Blood samples were collected from 1732 Tibetan sheep between February 2013 and April 2014 in Tianzhu county (Tianzhu) and Maqu county (Maqu) in Gansu province, northwestern China. Tianzhu (36°31′-37°55’N, 102°07′-103°46’E) and Maqu (33°06′-34°30’N, 100°45′-102°29’E) lie on the Tibetan Plateau. The average height of the two areas is 3000 meters above sea level, and both have a typical plateau continental climate, annual average temperature of −8 to +4°C. All samples were selected randomly, and different farms were chosen in different seasons. All the Tibetan sheep were farmed under semi-extensive conditions which mean that during day time sheep were grazed in communal natural grasslands and returned to fenced areas at night. Biometric data for Tibetan sheep, including age, breed and numbers of past pregnancies were obtained from the farmers and the study adhered to the highest standard (best practice) of veterinary care. Detailed information about pregnancies, source and age, and other characteristics was summarized. Blood samples were centrifuged at 2000 × *g* for 5 min and then sera were collected and stored at −20°C for further analysis.

### Serological examination

Serum samples from Tibetan sheep were diluted two-fold from 1:25 to 1:3200 and examined for *T. gondii* antibodies using the modified agglutination test (MAT) as described previously [8]. In this study, sera with MAT titers of 1:25 or higher were considered positive [3]. Positive and negative control sera were incorporated into each test. The positive control sera were collected from sheep experimentally infected with *T. gondii.* The positive and negative sera were provided by Prof Delin Zhang as a gift. The negative control sera were collected from sheep without *T. gondii* infection (collected before experimental infection and the negative sera were confirmed by IHA).The seronegative sheep were used for preparing positive sera according to the following protocol: the sheep were injected with 40 mg Excreted/Secreted Antigens twice with 2 weeks interval, and infected with 10^7^ tachyzoites of the RH strain two weeks later after the last immunization. The positive sera were collected 3 weeks later after infection. This infection experiment was approved by the Animal Ethics Committee of Lanzhou Veterinary Research Institute, Chinese Academy of Agricultural Sciences (Approval No. LVRIAEC2011-011). Those sera with questionable results were re-tested.

### Statistical analysis

To identify possible risk factors associated with exposure to *T. gondii* infection, a multivariable logistic regression analysis was carried out using the PASW Statistics 18.0 (SPSS Inc., IBM Corporation, Somers, NY). The risk factors included age (4 groups: between 0 and 1 year, between 1 and 3 years, between 3 and 5 years, and older than 5 years), gender (male and female), season (Spring, Summer, Autumn and Winter), geographical origin (Maqu and Tianzhu), and numbers of past pregnancies (four groups: 0, 1, 2, and more than 3 times). When independent variables was included in the multivariable logistic regression model and probability (*P*) value < 0.05 was considered as statistically significant between levels within factors and interactions, and their Odd ratio (OR) and 95% confidence interval (CI) were calculated. Differences in *T. gondii* seroprevalence among independent variables were analyzed by a Chi square test.

## Results

In this study, 352 (20.3%, 95% CI = 18.43-22.22) of 1732 Tibetan sheep were positive for antibodies against *T. gondii*, and the titers ranged from 1:25 to more than 1:3200 (titers of 1:25 in 191 samples, 1:50 in 64 samples, 1:100 in 41 samples, 1:200 in 6 samples, 1:400 in 8 samples, 1:800 in 6 samples, 1:1600 in 4 samples, and 1:3200 or higher in 32 samples). The seroprevalence of Tibetan sheep in Tianzhu and Maqu were 24.7% (95% CI = 21.63-27.72) and 16.8 (95% CI = 14.48-19.21), respectively. Positive samples were found in all four age groups, varied from 18.6 to 21.2%, and the highest prevalence was detected in Tibetan sheep of the between 1 and 3 years of age (21.2%, 95% CI = 17.89-24.51). The prevalence in females (19.2%, 95% CI = 17.01-21.46) were lower than in males (22.8%, 95% CI = 19.22-26.36). The numbers of parturition of female Tibetan sheep ranged between 0 pregnancy and above 3 pregnancies, and the *T. gondii* seroprevalence varied in female Tibetan sheep with different numbers of pregnancies, ranging from 19 to 21.5% (Table 1) with the highest seroprevalence (21.5%, 95% CI = 17.03-25.87) in 3 or higher pregnancies group. The seroprevalence in different season ranged from 16.5% in winter to 23.6% in summer (Table 1).

**Table 1:** **Seroprevalence of** ***Toxoplasma gondii*** **infection in Tibetan sheep in Gansu Province, northwest China by modified agglutination test (MAT)**

**Factor**	**Category**	**No. tested**	**No. positive**	**Prevalence (%)**	**95% CI**	**p-value** ^**a**^
Regions	Maqu	770	190	24.7	21.63-27.72	P < 0.001
	Tianzhu	962	162	16.8	14.48-19.21	
Gender	Male	531	121	22.8	19.22-26.36	P = 0.052
	Female	1201	231	19.2	17.01-21.46	
Season	Spring	480	108	22.5	18.76-26.24	P = 0.036
	Summer	398	94	23.6	19.45-27.79	
	Autumn	479	88	18.4	14.90-21.84	
	Winter	375	62	16.5	12.77-20.29	
Age (yr)	0 < yr ≤ 1	335	70	20.9	16.54-25.25	P = 0.729
	1 < yr ≤ 3	585	124	21.2	17.89-24.51	
	3 < yr ≤ 5	515	96	18.6	15.28-22.00	
	5 < yr	297	62	20.9	16.25-25.50	
Pregnancies	0	432	82	19.0	15.28-22.68	P = 0.545
	1	186	36	19.4	13.68-25.03	
	2	252	42	16.6	12.07-21.27	
	≥3	331	71	21.5	17.03-25.87	
Total		1732	352	20.3	18.43-22.22	

^a^P values were calculated using Chi square test.

Forward stepwise logistic regression analysis was employed to evaluate the risk factors of *T. gondii* infection in Tibetan sheep, and the results indicated that age, gender, and numbers of past pregnancies of Tibetan sheep were not a significant risk factors (*P* >0.05) and left out of the final model, however, season and geographical origin were considered as risk factors for the infection. The risk of acquiring the *T. gondii* infection in Tianzhu, where were 1.64 times higher (OR =1.637, 95% CI =1.291-2.075, *P* < 0.001) compared to Maqu. Samples collected in summer had a 1.61 times (OR =1.608, 95% CI =1.122-2.303, *P* = 0.010) higher risk of being seropositive compare to samples collected during winter, and had a 1.42 times (OR =1.419, 95% CI =1.002-2.011, *P* =0.049) higher risk of being seropositive compared to samples collected in spring, however, there was no statistically significant different between samples collected in winter or autumn (*P* > 0.05) (Table 2).

**Table 2:** **Odds ratios for geographical origin and season of Tibetan sheep as risk factors for** ***Toxoplasma gondii*** **in Tibetan sheep (n =1732)**

**Factor**	**Category**	**Prevalence (%)**	**OR**	**95% CI**	**P-value**
Region	Tianzhu	16.8	-	-	-
	Maqu	24.7	1.637	1.291-2.075	P < 0.001
Season	Winter	16.5	-	-	-
	Autumn	18.4	1.113	0.777-1.594	P = 0.559
	Summer	23.6	1.608	1.122-2.303	P = 0.010
	Spring	22.5	1.419	1.002-2.011	P = 0.049

## Discussion

*T. gondii* infection poses a great health hazard to both humans and animals. To date, a number of studies have been conducted on the seroprevalence of *T. gondii* infection in sheep from various geographical regions in the world, and the positive rates ranged from 3% to 95% [3]. One study reported that 121 (29.9%) of the 405 sheep exhibited antibodies against *T. gondii* by MAT in Michoacán State, Mexico [9], and Cenci-Goga *et al.* detected a 34.0% prevalence from 630 milk sheep by indirect immunofluorescence antibody test (IFAT) in Italy [10]. Lopes *et al.* found that 52.0% of 488 sheep were positive to *T. gondii* antibody tested by IFAT in São Paulo State, Brazil [11], and 33.6% of 119 sheep were infected by *T. gondii* detected by MAT in Portugal [12], The overall seroprevalence of *T. gondii* infection was 31.59% in sheep in East and West Shewa Zones of Oromia Regional State, Central Ethiopia [13]. These studies included both developed and developing countries but a common finding was that there was no correlation between the seroprevalence and economic development of different regions and countries. In this study, the overall *T. gondii* seroprevalence in Tibetan sheep in Gansu Province was 20.3%, which was lower than those in the above mentioned studies but higher than that observed in sheep in Borno state, Nigeria (6.7%) [14] and in Humid Pampa, Argentina (17.3%) [15]. In China, *T. gondii* seroprevalence in Tibetan sheep in Gansu was lower than that in Qinghai Province (29.8%) (although in some parts of Qinghai the *T. gondii* seroprevalence were quite high, such as in Nangqian County 39.4% and Zaduo County 48.7%) [6], but considerably higher than that observed previously in Tibet (5.7%) [7], and in domestic sheep in Liaoning Province (4.4%) [5]. Compared to other Asian countries, we found that *T. gondii* seroprevalence in Tibetan sheep in Gansu was higher than that in Rahim Yar Khan (Punjab), Pakistan (11.2%) and India (3.8%) [16,17], but lower than that reported in Iran (21.74%) [18]. The differences could be related to differences in ecological and geographical factors such as temperature, rainfall or landscape differences. The study area had overall low temperatures and it generally thought that the prevalence and risk of *T. gondii* infection decrease with decreasing temperature because it affect the survival of oocysts in the environment such as pastures.

The different sensitivity and specificity of the serological methods used to determine *T. gondii* prevalence may also be a factor contributing to the observed differences. Tibetan sheep in Maqu had a higher seroprevalence compared to sheep in Tianzhu, and the percentage of positive samples with high titers (1:400 or higher) in Ganan was higher than in Tianzhu. The proportion of sheep with high titers was higher in Ganan compared to Tianzhu which could be due to a more recent infection or a higher parasite inoculum. The ecological environment of Ganan and Tianzhu are similar, so ecological environment may not be a factor responsible of the differences in seroprevalence. The difference could cause by the hygiene conditions or other factors for instance the number of cats in the two areas. Sheep acquire *T. gondii* infection mainly by ingestion of oocysts from the environment and congenital transmission. Whereas all the studied Tibetan sheep were farmed under semi-extensive conditions and had good opportunity to ingest oocysts from pastures, which may explain the high seroprevalence of *T. gondii* infection in this study.

The final logistic regression model showed that age was not the significant risk factor associated with exposure to *T. gondii* infection. Although the highest (21.2%, 95% CI = 17.89-24.51) seroprevalence found in the age group between 1 and 3 years, and the univariate analysis showed that the difference was not significant (*P* >0.05). Thus, there was no correlation between the seroprevalence and age in Tibetan sheep. This result indicated that age was not a crucial risk factor for *T. gondii* infection in Tibetan sheep in Gansu province, which was similar to previous studies [7,9]. However, there were some studies indicated that seroprevalence of *T. gondii* increased with age [12,19], and those results were different with our study and other studies. We had expected that age was an important factor for being seropositive as a measure of the cumulated life-time risk. The climatic condition in the area is extreme with low temperatures in both summer and winter due mainly to the altitude. It is possible that this influence the risk of infection in the sheep and this needs further studies for instance by sampling the pastures for *T. gondii* oocysts. The lambs arrive in summer when it is warmest and they are immediately exposed to infection on the pastures. If the risk of infection the rest of the year is very limited due to the low temperatures, risk of infection could be limited to a few months every year, thus infection is an early event which may – at least partly – explain the lack of correlation with age.

With regard to gender, the prevalence in males was higher than in females, but the difference was not statistically significant, which is in agreement with the conclusions of one study [9]. However, several studies indicated that the prevalence in females were higher than males [11,14,20], which was probably due to the lower levels in immune response or antibody persistence of females in some periods of their lives.

In our study, of the 1201 female Tibetan sheep examined, 769 had at least one previous pregnancies. The seroprevalence in the Tibetan sheep with ≥3 births was the highest (21.5%, 95% CI = 17.03-25.87), followed by the Tibetan sheep with 1 pregnancy, there was no statistically significant difference in seroprevalence between sheep of different parity. The results of the present study clearly revealed that the season was a crucial risk factors associated with *T. gondii* seroprevalence. In this study, we found that the seroprevalence in Tibetan sheep were higher in summer and in spring, they had a 1.61 times and 1.42 times higher risk of being infection compared to Tibetan sheep in winter. The temperature and the range of cats may be explaining the significant seasonal differences in prevalence. In spring and summer, the climate is warm and damp which are favorable for the survival of *T. gondii* oocysts, in addition, cats are more active at warm temperature and expand their range which lead to oocysts widely distribution. These may contribute to the higher seroprevalence in spring and summer.

This is the first report of *T. gondii* infection and likely factors associated with *T. gondii* infection in Tibetan sheep in Gansu Province, northwestern China, where mutton is the most liked meat by the local people, and the Tibetan sheep populations was large enough for the results to be conclusive. However, to better understand risk factors associated with *T. gondii* infection in this region, further studies are required that provide detailed information on risk factors associated with *T. gondii* infection, such as production system, Tibetan sheep welfare, food source and the number of cats.

## Conclusions

The results of the present survey showed a high seroprevalence of *T. gondii* in Tibetan sheep in Gansu province, northwestern China, and geographical origin and season are main risk factors associated with *T. gondi*i infection. The higher seroprevalence of *T. gondii* infection in spring and summer may provide seasonal information to prevent and control *T. gondii* infection in Tibetan sheep, which could help reduce *T. gondii* infection in humans.
